# Therapeutic efficacy of denosumab for rheumatoid arthritis: a systematic review and meta-analysis

**DOI:** 10.1093/rap/rkab099

**Published:** 2021-12-17

**Authors:** Mayu Yagita, Takayoshi Morita, Atsushi Kumanogoh

**Affiliations:** 1 Department of Respiratory Medicine and Clinical Immunology, Osaka University Graduate School of Medicine; 2 Laboratory of Immunopathology, World Premier International Immunology Frontier Research Center; 3 Integrated Frontier Research for Medical Science Division, Institute for Open and Transdisciplinary Research Initiatives, Osaka University; 4 Center for Infectious Disease for Education and Research (CiDER), Osaka University, Suita, Osaka, Japan

**Keywords:** denosumab, bone mineral density, bone erosion, meta-analysis, rheumatoid arthritis

## Abstract

**Objectives:**

Denosumab is used for osteoporosis because it inhibits osteoclast maturation and suppresses bone resorption. Although denosumab is expected to inhibit the bone erosion in RA, its therapeutic efficacy is not well established. The aim of this study was to estimate the effects of denosumab on RA through a meta-analysis.

**Methods:**

A systematic review was conducted in accordance with the Preferred Reporting Items for Systematic Reviews and Meta-Analyses. PubMed, Web of Science and Scopus were searched for original studies providing information on BMD, joint destruction and disease activity in denosumab-treated RA. A random-effects model was used in the meta-analysis.

**Results:**

Of the 367 studies identified, 18 met the selection criteria. The BMDs of the lumbar spine, total hip and femoral neck at 12 months after denosumab treatment increased by 5.27% (95% CI: 4.37, 6.18), 2.82% (2.46, 3.18) and 3.07% (2.66, 3.48), respectively. In the sensitivity analysis, age and sex tended to influence the effect of denosumab therapy on the rate of variation of BMD, but not glucocorticoid use. The changes in the modified total sharp, erosion and joint space narrowing scores at 12 months after denosumab treatment were significantly smaller with denosumab than with placebo, although the DAS did not change after denosumab treatment.

**Conclusion:**

Although denosumab has an inhibitory effect on the bone resorption in RA, its effects might be influenced by the age and sex of RA patients, but not by glucocorticoid use.

Key message

- Denosumab is an interesting drug that can inhibit bone destruction in RA without immunosuppression.

## Introduction

RA is a chronic inflammatory autoimmune disease characterized by synovitis and joint cartilage destruction [1]. Continuous synovitis causes joint and bone destruction, resulting in joint dysfunction and poor quality of life. Therefore, prevention of the progression of joint destruction in synovitis is crrucially important for patients with RA. Recently, biologics, such as TNF-α or IL-6 receptor blockers, are broadly used for RA to suppress inflammation and bone erosion [2]. Although anti-inflammatory treatments dramatically affect RA, ∼5% of patients with RA have sustained synovitis and show progression of bone destruction despite biologic treatment or when biologics are difficult to use because of susceptibility to infection or drug side effects [3, 4].

Denosumab (DMB) is a fully human monoclonal anti-RANK ligand (anti-RANKL) antibody that inhibits the differentiation and maturation of osteoclasts by the binding of RANKL to RANK. Thus, DMB can powerfully suppress the osteolytic function of osteoclasts and increase BMD in patients with osteoporosis [5]. DMB increased BMD persistently in post-menopausal women with osteoporosis for 10 years and decreased their risk of fracture [6]. In patients with glucocorticoid (GC)-induced osteoporosis, it also increased the BMDs of the lumbar spine and total hip more than did bisphosphonates [7].

Recently, DMB has been suggested to improve modified total sharp scores (mTSSs) and modified sharp erosion scores (ESs) in clinical studies [8, 9]. However, DMB might have no effect on the disease activity of RA [8, 9]. The evidence is insufficient to prove whether DMB can suppress bone erosion and increase BMD in patients with RA in the inflammatory conditions inducing osteoclastogenesis. Some recommendations for RA, including the EULAR recommendations, do not mention anti-RANKL antibody therapy for RA [2].

To understand the effects of DMB on BMD, mTSS, ES, joint space narrowing score (JSNS), DAS28-ESR, simple disease activity index (SDAI), bone turnover markers [type I procollagen N-terminal propeptide (P1NP) and tartrate-resistant acid phosphatase-5b (TRACP-5b)] and the risk of bone fracture and upper respiratory infection in patients with RA, we carried out a systematic review of original articles that reported such information in patients with RA treated with DMB and made a quantitative summary of the accumulated evidence on the efficacy of DMB for RA by performing a meta-analysis.

## Methods

### Registration

This meta-analysis was performed in accordance with the Preferred Reporting Items for Systematic Reviews and Meta-analyses (PRISMA) statement (Supplementary File S1, available at *Rheumatology Advances in Practice* online) [10] and registered with PROSPERO (CRD4202193268).

### Search strategy

Articles documenting the effects of DMB on the BMD, joint destruction, disease activity or the risk of fracture and infection in patients with RA were examined using three websites (PubMed, Web of Science and Scopus), which were searched using [denosumab AND ‘rheumatoid arthritis’]. No language restrictions were applied. The search was performed three times to identify articles published between January 2000 and August 2021. Final searches were performed on 17 August 2021.

### Article selection process

The primary screening was performed as follows. We included original articles (excluding reviews and case reports) and human research; title or abstract, including the term ‘rheumatoid arthritis’ and ‘denosumab’; available on the Internet and linkage from the search site to the full text of the article. In the secondary screening, studies that provided no raw data on the mean (S.d.) of the difference in the rates of the changes in BMD, mTSS, ES, JSNS, P1NP, TRACP-5b, DAS28-ESR and SDAI at 12 months after administration of DMB were excluded. Redundancies between PubMed, Web of Science and Scopus were eliminated; that is, individual studies were counted only once in this analysis. We considered patients with RA in each study as those diagnosed on the basis of the 1987 revised ACR criteria or 2010 ACR/EULAR classification system [11, 12].

### Quality assessment

Two authors (M.Y. and T.M.) independently checked and selected all the references. When the results were inconsistent, a third person (A.K.) provided an opinion to resolve the issue. The quality of the selected studies was assessed using the Study Quality Assessment Tools (Quality Assessment Tool for Observational Cohort and Cross-Sectional Studies) from the National Heart, Lung, and Blood Institute [13]. Funnel plot asymmetry was used to assess publication bias.

### Data extraction

Data were extracted from all studies included in this analysis [author, year of publication, number of patients, percentage of females, disease duration of RA, BMI, positivity of RF and ACPA, DAS28-CRP, DAS28-ESR, SDAI, CRP, HAQ-disability index (HAQ-DI), percentage of patients treated with prednisolone (PSL)/MTX/biologic DMARDs (bDMARDs), BMD of the lumbar spine, total hip or femoral neck, mTSS, ES, JSNS, P1NP, TRACP-5b and number of bone fractures] and entered into Table 1 and Supplementary Table S1, available at *Rheumatology Advances in Practice* online. To evaluate the effects of DMB in the patients with RA, the mean (S.d.) of the rates of the changes in BMD, mTSS, ES, JSNS, P1NP, TRACP-5b, DAS28-ESR and SDAI, the number of bone fractures at 12 months after the start of treatment with DMB, placebo or bisphosphonates (BPs), and the number of patients with upper respiratory infection during observational periods between the DMB group and placebo group were extracted. When raw data were unavailable, we calculated the values manually using information available in the published graphs. In the studies reporting only the median and interquartile range or range, the mean (S.d.) was calculated using the methods of Wan *et al.* [31].

**Table 1:** Characteristics of included studies in this meta-analysis

Authors (year)	Study design	Group	Number of subjects	Age (years), mean (S.d.)	Female (%)	Duration (years), mean (S.d.)	BMI (kg/m^2^), mean (S.d.)	RF (%)	ACPA (%)	DAS28-CRP, mean (S.d.)	GC (%)	MTX (%)
Mochizuki *et al.* (2021) [14]	Cohort	DMB	112	75 (68, 81)^a^	NA	13 (7, 24)^a^	NA	77.7	77.7	NA	42	55.4
Mori *et al.* (2021) [15]	Cohort	BPs	50	67.6 (7.4)	NA	9.7 (5.1)	23.4 (4.4)	NA	NA	3.1 (1.1)	44	76
		DMB	56	71.1 (8.2)	NA	11.3 (5.2)	22.8 (3.9)	NA	NA	2.8 (1.2)	32.1	46.4
So *et al.* (2021) [16]	RCT	Placebo	55	56.5 (7.1)	86	6.1 [3.5–11.5]^b^	NA	72	78	2.4 (0.8)	11	76
		DMB	55	57.2 (8.5)	75	4.4 [2.4–10.7]^b^	NA	69	80	2.6 (0.9)	11	87
Tanaka *et al.* (2021) [17]	RCT	Placebo	105	54.5 (12.6)	77.1	2.3 (1.4)	NA	59	67.6	3.4 (1.0)	30.5	65.7
		DMB	191	57.2 (12).1	78.5	2.3 (1.3)	NA	63.4	71.2	3.6 (1.1)	31.9	56.5
Tanaka *et al.* (2020) [18]	RCT	Placebo	306	56.2 (11.4)	79.4	2.1 (1.3)	NA	64.4	69	NA	34.6	90.8
		DMB	302	57.0 (11).9	77.2	2.2 (1.3)	NA	65.9	75.5	NA	36.1	86.4
Hattori *et al.* (2019) [19]	Cohort	DMB	74	70.2 (7.6)	NA	17.1 (12.8)	20.0 (3.1)	NA	NA	2.8 (1.2)	35.1	64.9
Ishiguro *et al.* (2019) [20]	RCT	Placebo	66	57.0 (10. 6)	86.4	NA	NA	NA	NA	NA	NA	NA
		DMB	70	54.4 (10.6)	76.5	NA	NA	NA	NA	NA	NA	NA
Kaneko *et al.* (2019) [21]	Cohort	Naïve	25	66.2 (1.6)	NA	13.2 (2.0)	NA	68	72	NA	32	60
		Switched	11	75.4 (2.2)	NA	15.5 (2.7)	NA	7.27	81.8	NA	54.5	45.5
Suzuki *et al.* (2019) [22]	Cohort	DMB	31	68.7 (1.2)	NA	NA	20.1 (0.6)	NA	NA	3.1 (0.2)	NA	NA
		DMB + VD	27	68.3 (1.7)	NA	NA	20.4 (0.4)	NA	NA	3.0 (0.2)	NA	NA
Takeuchi *et al.* (2019) [23]	RCT	Placebo	218	55.8 (11.7)	76.6	2.1 (1.3)	22.7 (3.5)	62.8	66.5	3.4 (1. 0)	31.7	87.2
		DMB	217	58.1 (12.3)	77.4	2.2 (1.3)	22.4 (3.7)	64.5	72.8	3.6 (1.1)	33.6	81.1
Ebina *et al.* (2018) [24]	Cohort	BPs	30	67.6 (1.8)	100	18.3 (1.9)	22.2 (0.6)	NA	90	2.5 (0. 2)	60	76.7
		DMB	30	68.5 (1.8)	100	18.2 (2.4)	20.5 (0.6)	NA	86.7	2.2 (0.2)	66.7	86.7
Ebina *et al.* (2018) [25]	Cohort	BPs	80	66.2 (1.0)	100	17.9 (1.1)	NA	82.5	78.8	2.3 (0.	72.5	66.3
		DMB	74	66.4 (1.5)	100	18.5 (1.6)	NA	77	75.7	1) 2.1 (0.1)	74.3	62.2
Mochizuki *et al.* (2018) [26]	Cohort	DMB	70	70.9 (8.8)	NA	15.0 (11.6)	NA	77.1	72.9	2.2 (0.7)	NA	NA
Nakamura *et al.* (2017) [27]	Cohort	DMB	22	70.9 (1.8)	100	16.6 (2.9)	20.8 (0.9)	NA	NA	3.1 (0.3)	NA	NA
		DMB + VD	21	70.6 (2.3)	100	18.5 (1.6)	20.0 (0.9)	NA	NA	3.0 (0.3)	NA	NA
Nakamura *et al.* (2017) [28]	Cohort	BPs	26	69.8 (1.3)	NA	15.3 (2.2)	20.8 (0.6)	NA	NA	3.5 (0.3)	NA	NA
		DMB	26	70.6 (1.9)	NA	15.5 (2.9)	20.8 (0.6)	NA	NA	3.8 (0.3)	NA	NA
Kinoshita *et al.* (2017) [29]	Cohort	BPs	49	67.7 (10.0)	93.9	13.2 (11.4)	NA	NA	NA	NA	NA	NA
		DMB	49	70.6 (7.6)	95.9	12.4 (9.8)	NA	NA	NA	NA	NA	NA
Takeuchi *et al.* (2016) [9]	RCT	Placebo	88	57.0 (10.6)	86.4	2.3 (1.3)	22.1 (3.3)	68.2	75	4.0 (1.0)	42	NA
		DMB	85	54.4 (10.6)	76.5	2.2 (1.3)	22.3 (3.7)	69.4	82.4	3.6 (1.0)	42.4	NA
Dore *et al.* (2010) [30]	Cohort	Placebo_nb	56	55.7 (11.9)	79	9.9 (8.2)	NA	80	NA	NA	NA	NA
		DMB_nb	60	55.5 (11.0)	70	9.9 (7.0)	NA	72	NA	NA	NA	NA
		Placebo_b	22	60.4 (8.1)	82	9.3 (8.0)	NA	73	NA	NA	NA	NA
		DMB_b	13	65.4 (10.3)	69	13.2 (7.7)	NA	92	NA	NA	NA	NA
		Placebo_ng	49	57.7 (10.2)	86	10.6 (8.7)	NA	78	NA	NA	0	NA
		DMB_ng	45	59.4 (10.5)	78	10.2 (6.8)	NA	76	NA	NA	0	NA
		Placebo_g	29	55.8 (12.7)	69	8.2 (6.9)	NA	79	NA	NA	100	NA
		DMB_g	29	53.9 (12.3)	57	10.8 (7.8)	NA	75	NA	NA	100	NA
Cohen *et al.* (2008) [8]	Cohort	Placebo	78	57.0 (11.1)	79	9.7 (8.1)	NA	78	NA	NA	36	NA
		DMB	73	57.3 (11.4)	70	10.5 (7.2)	NA	75	NA	NA	37	NA

a Median (interquartile range: Q1, Q3). ^b^95% CI. b: bisphosphonates; BPs: bisphosphonates; DMB: denosumab; g: glucocorticoids; GC: glucocorticoid; NA: not available; nb: no bisphosphonates; ng: no glucocorticoids; RCT: randomized controlled trial; VD: vitamin D.

### Data synthesis

A meta-analysis was performed to estimate the efficacy of DMB for RA. Clinical data were analysed before and after DMB treatment or between DMB and placebo or BPs. The outcomes were expressed as the mean or mean differences and 95% CIs by using the random-effects model. Heterogeneity was assessed by the *I*^2^ test, values of *I*^2^ of 25, 50 and 75% were defined as low, moderate and high, respectively [32]. All the analyses were conducted using R v.3.5.1 (R project for Statistical Computing) and EZR v.1.29 [33].

### Sensitivity and subgroup analyses

Sensitivity analyses were performed using age, sex, disease duration of RA, positivity of RF and ACPA and the utilization rates of GC and MTX. The Spearman correlation coefficient was calculated using R v.3.5.1. Values of *P* ≤ 0.05 were considered statistically significant. Subgroup analyses were performed using age, disease duration of RA and GC usage.

## Results

### Study selection

**Fig. 1:**
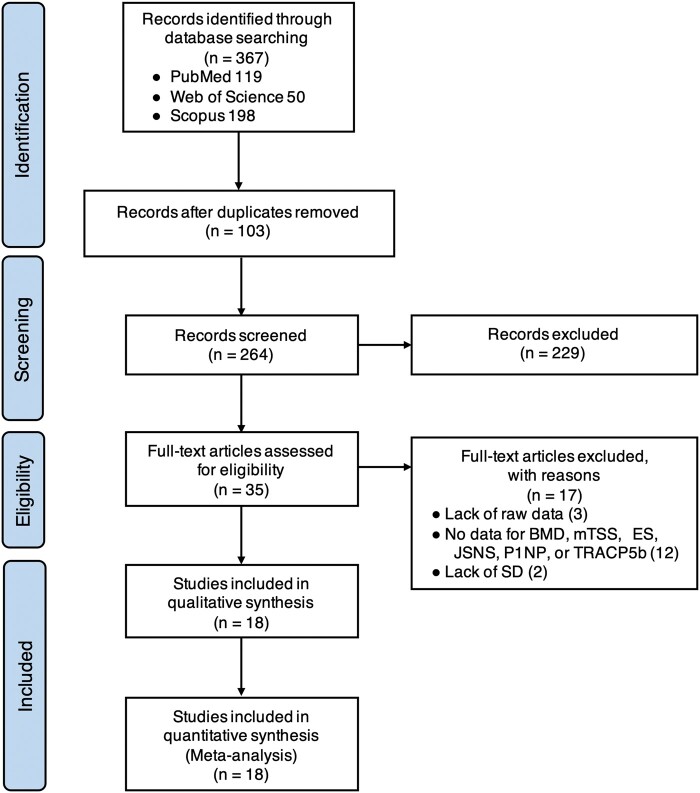
PRISMA 2009 flow diagram ES: erosion score; JSNS: joint space narrowing score; mTSS: modified total sharp score; P1NP: type I procollagen N-terminal propeptide; TRACP-5b: tartrate-resistant acid phosphatase-5b.

This study identified 367 records from three search sites (119, 50 and 198 studies on PubMed, Web of Science and Scopus, respectively), of which 103 were excluded because of duplications between the three search sites, 229 were removed by primary screening, and 17 records were removed for the following reasons: lack of raw data (3), no data on mean BMD, mTSS, ES, JSNS, P1NP, TRACP-5b, DAS28-ESR and SDAI (12); and no data on S.d. (2). Finally, 18 studies [8, 9, 14–30] met the selection criteria and were included in the meta-analysis (Fig. 1).

### Characteristics of the studies and patient backgrounds

The total of 1696 patients with RA who were treated with DMB and 1222 patients with RA who were treated with placebo or BPs were enrolled. The characteristics of the patients with RA at baseline (before DMB treatment) in each selected study in this meta-analysis are summarized in Table 1 and Supplementary Table S1, available at *Rheumatology Advances in Practice* online. Six studies were randomized controlled trials (RCTs), and 12 were cohort studies. The ages of the patients ranged from 53.9 to 75.4 years; percentage of females, 57–100%; disease duration, 2.1–18.3 years; BMI, 20.0–23.4 kg/m^2^; positivity of RF, 59–92%; positivity of ACPA, 66.5–90%; HAQ-DI, 0.125–0.9; DAS28-CRP, 2.1–4.0; DAS28-ESR, 2.8–4.2; SDAI, 4.3–21.3; CRP, 0.15–1.10 mg/dl; rate of GC use, 11–100%; amount of GC, 2.8–5.4 mg/day; and rate of MTX use, 45.5–90.8%. The patients with RA who were treated with placebo in seven [8, 9, 16–18, 20, 23] out of eight studies had received the medication of calcium and vitamin D.

The total score in the study quality assessment tool from the National Heart, Lung, and Blood Institute ranged from 8 to 14 in each study (Supplementary Table S2, available at *Rheumatology Advances in Practice* online). The results of the funnel plots indicated possibly some publication biases or systemic heterogeneities in the analysis of the BMD of the lumbar spine at 12 months (Supplementary Fig. S1, available at *Rheumatology Advances in Practice* online).

### Effect of DMB on BMD in comparison to placebo and BPs in patients with RA

**Fig. 2:**
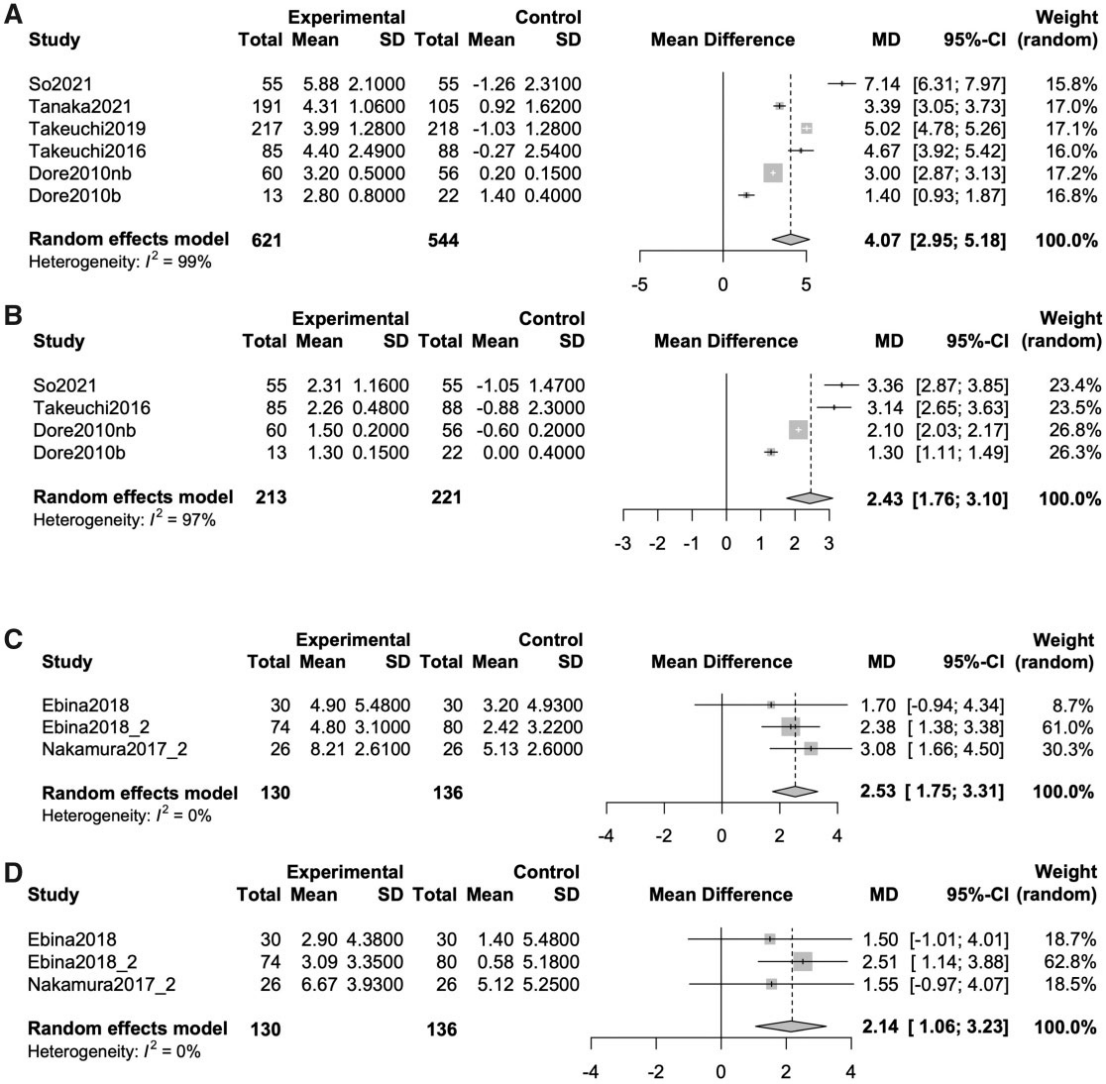
Forrest plot of mean difference in BMD at 12 months from baseline between denosumab and placebo or bisphosphonates (**A**) Mean difference of BMD in lumber spine at 12 months from baseline between denosumab and placebo. (**B**) Mean difference of BMD in total hip at 12 months from baseline between denosumab and placebo. (**C**) Mean difference of BMD in lumber spine at 12 months from baseline between denosumab and BPs. (**D**) Mean difference of BMD in total hip at 12 months from baseline between denosumab and BPs. BPs: bisphosphonates; MD: mean difference.

The variation rate of BMD in the lumbar spine and total hip at 12 months after DMB treatment was evaluated in comparison to that after placebo treatment. Five studies [16, 17, 23, 30, 33] and six groups (number of patients: DMB, 621; and placebo, 544) at the lumbar spine, and three studies [9, 16, 30] and four groups (DMB, 213; and placebo, 221) at the total hip were selected. The mean differences in BMD of the lumbar spine and total hip between DMB and placebo were 4.07% (95% CI: 2.95, 5.18; *P* < 0.0001, *I*^2^ = 99%) and 2.43% (1.76, 3.10; *P* < 0.0001, *I*^2^ = 97%), respectively (Fig. 2A and B). The rates of variation of the BMDs of the lumbar spine and total hip at 12 months after DMB treatment were also evaluated and compared with those after BP treatment. Three studies [25, 26, 28] and three groups (number of patients: DMB, 130; and BPs, 136) at the lumbar spine and total hip were selected. The mean differences in the BMDs of the lumbar spine and total hip between DMB and BPs were 2.53% (95% CI: 1.75, 3.31; *P* < 0.0001, *I*^2^ = 0%) and 2.14% (1.06, 3.23; *P* < 0.0001, *I*^2^ = 0%), respectively (Fig. 2C and D). On the basis of these results, the BMDs of the lumbar spine and total hip in the patients with RA increased significantly at 12 months after treatment with DMB in comparison to placebo and BPs.

### Variation rates of BMDs between baseline and 12 months after DMB treatment in patients with RA

**Fig. 3:**
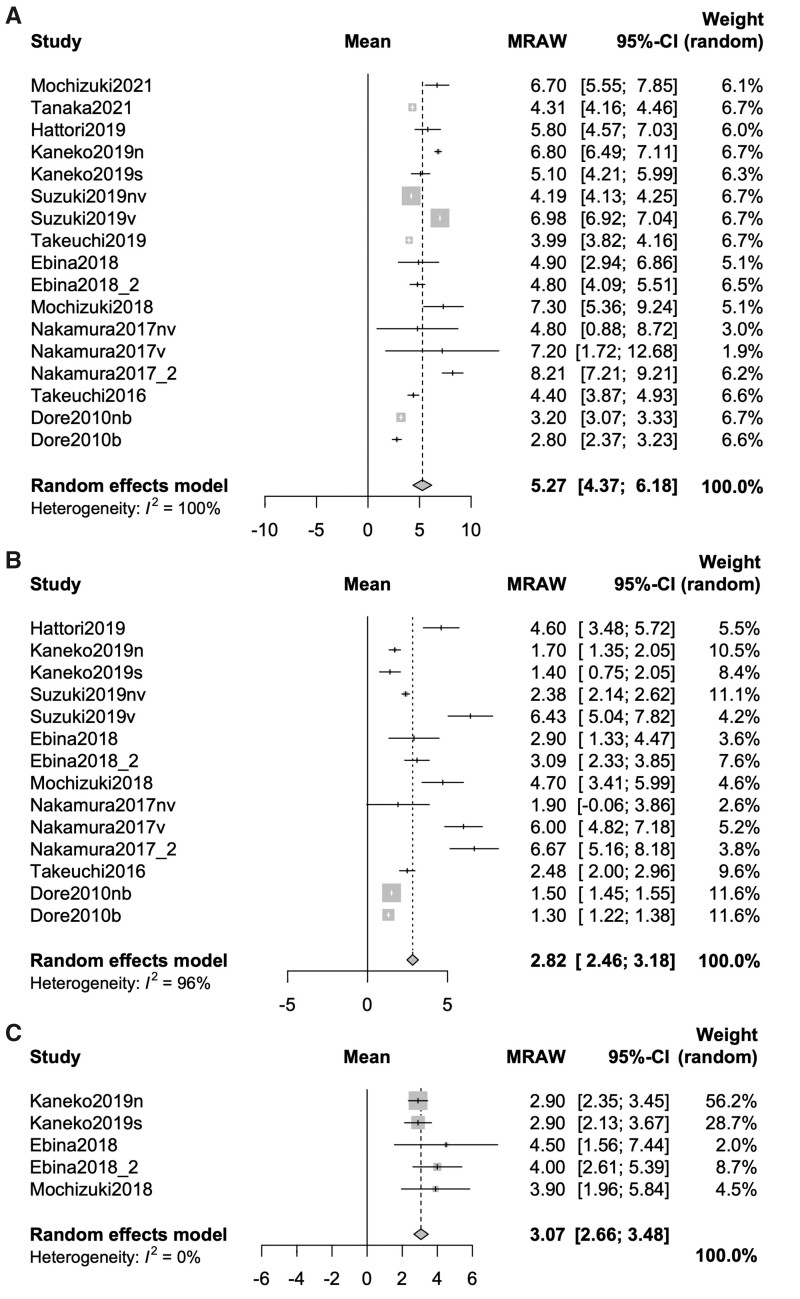
Forrest plot of the variation rate of BMD at 12 months after denosumab treatment (**A**) The variation rate of BMD in lumber spine. (**B**) The variation rate of BMD in total hip. (**C**) The variation rate of BMD in femoral neck. MRAW: mean raw value.

The variation rates of the BMDs of the lumbar spine, total hip and femoral neck between baseline and 12 months after DMB treatment in the patients with RA were evaluated. Fourteen studies [9, 14, 17, 19, 21–30] and 17 groups, 10 studies [9, 19, 21, 22, 24–30] and 14 groups, and 4 studies [21, 24–26] and 5 groups (number of patients: lumbar spine, 1089; total hip, 569; and femoral neck, 210) were selected. The combined variation rates of the BMDs of the lumbar spine, total hip and femoral neck were 5.27% (4.37–6.18; *P* < 0.0001, *I*^2^ = 100%), 2.82% (2.46–3.18; *P* < 0.0001, *I*^2^ = 96%) and 3.07% (2.66–3.48; *P* < 0.0001, *I*^2^ = 0%), respectively (Fig. 3).

### Sensitivity and subgroup analyses of the variation rates of the BMD of the lumbar spine and total hip after DMB treatment

Owing to the high heterogeneity in the meta-analysis of the variation rates of the BMD of lumbar spine and total hip between baseline and 12 months after DMB treatment, a sensitivity analysis was performed, focusing on age, sex, disease duration of RA, positivity of RF and rates of GC and MTX use. The variation rates of BMD in lumber spine exhibited significant positive correlations with age and sex (ρ = 0.617 and *P* = 0.008; and ρ = 0.909 and *P* < 0.001) and tended to exhibit positive correlations with disease duration (ρ = 0.489, *P* = 0.065) and the positivity of RF (ρ = 0.667, *P* = 0.083). The percentages of GC and MTX usage were not correlated with the variation rates of BMD (Supplementary Fig. S2A, available at *Rheumatology Advances in Practice* online). Likewise, the variation rates of BMD in total hip exhibited a significant positive correlation with sex (ρ = 0.808 and *P* = 0.028) and tended to exhibit a positive correlation with disease duration (ρ = 0.516, *P* = 0.086), but not age (Supplementary Fig. S2B, available at *Rheumatology Advances in Practice* online). The positivity of RF and the percentages of GC and MTX usage were not correlated with the variation rates of BMD in total hip (data not shown).

**Fig. 4:**
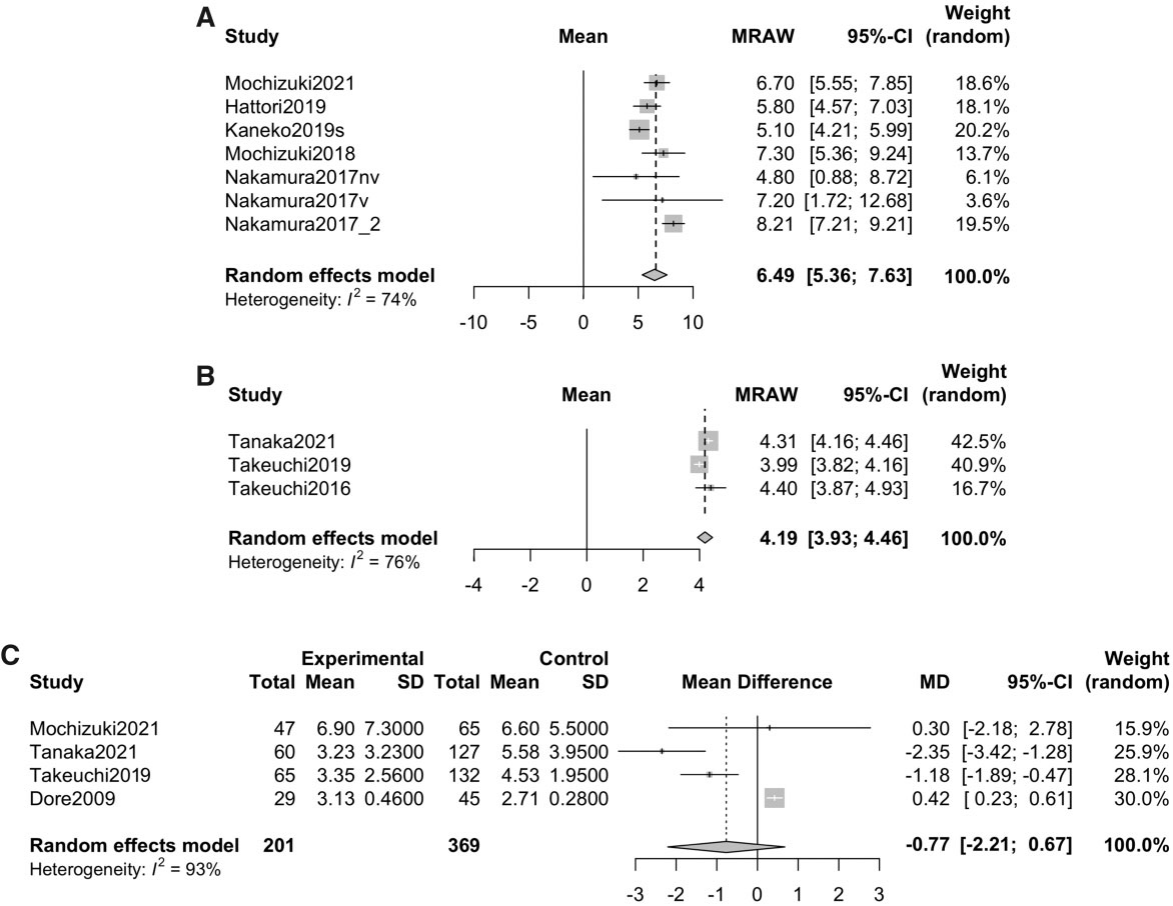
Forrest plot of subgroup analysis (**A**) The variation rate of BMD in lumber spine at 12 months after DMB treatment in the groups with a mean disease duration of ≥15 years since the onset of RA and a mean age of ≥70 years. (**B**) The variation rate of BMD in lumber spine at 12 months after denosumab treatment in the groups with a mean disease duration of <5 years since the onset of RA and a mean age of <60 years. (**C**) The mean difference of BMD in lumber spine at 12 months after denosumab treatment between patients with RA treated with GC and those treated without GC. GC: glucocorticoid; MRAW: mean raw value.

Following the results from the sensitivity analysis, subgroup analyses were performed with a focus on age, disease duration and GC usage. The variation rates of the BMD of the lumbar spine between baseline and 12 months after DMB treatment in the group [14, 16–28] with a mean duration of ≥15 years since the onset of RA and a mean age of ≥70 years were evaluated (6.49% [5.36–7.63]; *P* < 0.0001, *I*^2^ = 74%; Fig. 4A). Likewise, the variation rates of BMD in the group [9, 17, 23] with a mean duration of <5 years since the onset of RA and a mean age of <60 years were evaluated (4.19% [3.93–4.46]; *P* < 0.0001, *I*^2^ = 76%; Fig. 4B). The mean difference in the variation rate of the BMD of the lumbar spine at 12 months from baseline between patients with GC and those without GC was also evaluated (−0.77% [−2.21 to 0.67]; *P* = 0.296, *I*^2^ = 93%) [14, 17, 23, 30], and there was no difference in the variation rate of the BMD between them (Fig. 4C).

### Variation rates of bone metabolic markers between baseline and 12 months after DMB treatment in patients with RA

The variation rates of P1NP and TRACP-5b from baseline to 12 months after DMB treatment were evaluated. Seven studies [8, 14, 19, 24–27] and 8 groups (number of patients: 476) for P1NP, and 8 studies [14, 19, 22, 24–28] and 10 groups (number of patients: 487) for TRACP-5b were selected. The mean variation rates of P1NP and TRACP-5b between baseline and 12 months after DMB treatment were −36.34% (−45.98 to −26.69; *P* < 0.0001, *I*^2^ = 99%) and −35.49% (−46.04 to −29.94; *P* < 0.0001, *I*^2^ = 98%), respectively (Supplementary Fig. S3, available at *Rheumatology Advances in Practice* online).

### Variation rates of sharp scores for joint destruction between DMB and placebo at 12 months from baseline in patients with RA

**Fig. 5:**
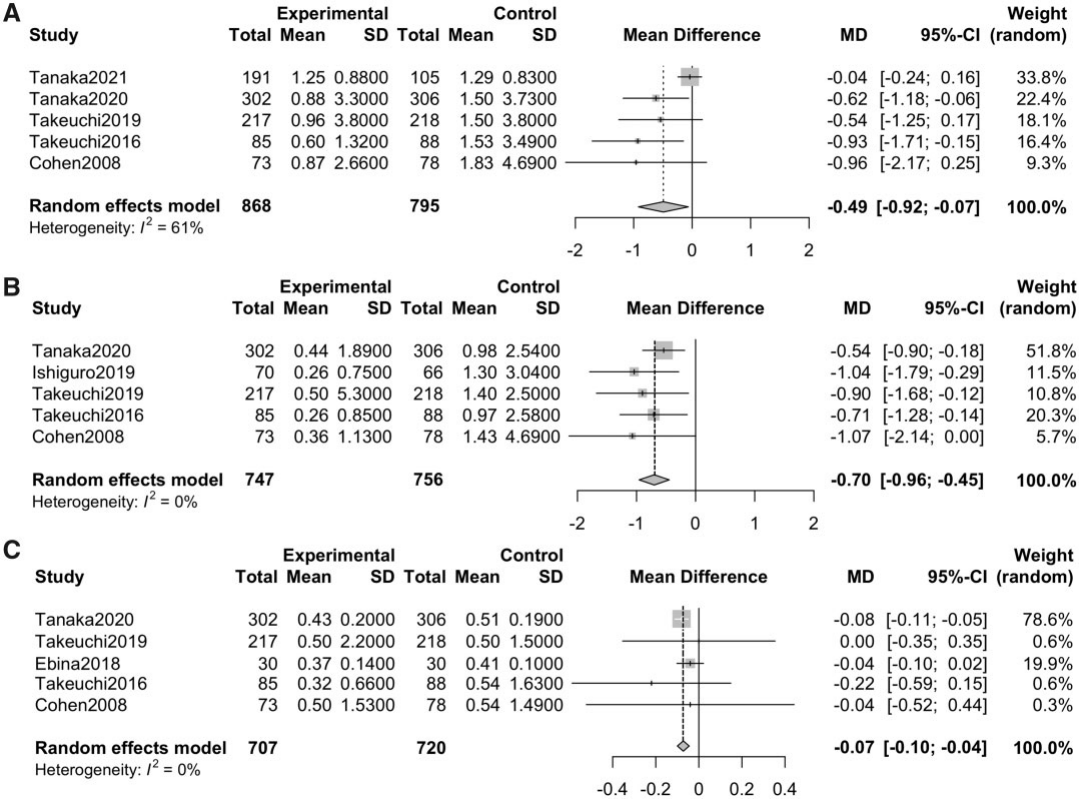
Forrest plot of modified total sharp score, erosion score and joint space narrowing score at 12 months from baseline between denosumab and placebo (**A**) Mean difference of mTSS at 12 months from baseline between denosumab and placebo. (**B**) Mean difference of ES at 12 months from baseline between denosumab and placebo. (**C**) Mean difference of JSNS at 12 months from baseline between denosumab and placebo. ES: erosion score; JSNS: joint space narrowing score; MD: mean difference; mTSS: modified total sharp score.

The variation rates of the mTSS, ES and JSNS between the group treated with DMB and that treated with placebo at 12 months from baseline were evaluated. Five studies [8, 9, 17, 18, 23] and five groups (number of patients: DMB, 868; and placebo, 795) for the mTSS, five studies [8, 9, 18, 20, 23] and five groups (number of patients: DMB, 747; and placebo, 756) for the ES, and five studies [8, 9, 18, 23, 24] and five groups (number of patients: DMB, 707; and placebo, 720) for the JSNS were selected. The mean differences in the mTSS, ES and JSNS between the DMB and placebo groups were −0.49% (−0.92 to −0.07; *P* = 0.024, *I*^2^ = 61%), −0.70% (−0.96 to −0.45; *P* < 0.0001, *I*^2^ = 0%) and −0.07% (−0.10 to −0.04; *P* = 0.007, *I*^2^ = 0%), respectively (Fig. 5). These results suggest that DMB has a suppressive function for joint destruction.

### Difference in the disease activity of RA at 12 months after DMB treatment in patients with RA

The differences in the DAS28-ESR and SDAI from baseline to 12 months after DMB treatment were evaluated. Three studies [21, 26, 29] and 4 groups (number of patients: 155) for the DAS28-ESR and SDAI were selected. The mean differences in the DAS28-ESR and SDAI between baseline and 12 months after DMB treatment were −0.04% (−0.12 to 0.04; *P* < 0.298, *I*^2^ = 0%) and 0.40% (−0.08 to 0.88; *P* < 0.102, *I*^2^ = 0%), respectively (Supplementary Fig. S4, available at *Rheumatology Advances in Practice* online). Following these results, there was no difference in the disease activity of RA between baseline and 12 months after DMB treatment.

### Evaluation of the fracture risk between DMB and BPs in patients with RA

The fracture risk between the group with DMB and the group with placebo or BPs was evaluated. Three studies [9, 17, 23] and three groups (number of patients: DMB, 411; and placebo, 493), and three studies [15, 24, 28] and three groups (number of patients: DMB, 156; and BPs, 156) were selected. There were no patients with fracture in the DMB group and the placebo group, and there was no difference in the fracture risk between DMB and placebo among patients with RA (data not shown). Likewise, the fracture risk between DMB and BPs at 12 months from baseline was evaluated (0.46 [0.15–1.45]; *P* = 0.185, *I*^2^ = 0%; Supplementary Fig. S5, available at *Rheumatology Advances in Practice* online).

### Evaluation of the risk of upper respiratory infection between DMB and placebo in patients with RA

The risk of upper respiratory infection between the group with DMB and the group with placebo was evaluated. Four studies [8, 9, 23, 34] and four groups (number of patients: DMB, 449; and placebo, 462) were selected. There was no difference in the risk of upper respiratory infection between DMB and placebo among patients with RA (1.06 [0.88–1.27]; *P* = 0.599, *I*^2^ = 0%; Supplementary Fig. S6, available at *Rheumatology Advances in Practice* online).

## Discussion

It is known that the percentage of patients with osteoporosis is about twice as high in patients with RA (29.9%) as in the general population (17.4%) [35]. The fracture risk in patients with RA has been reported to increase compared with that in patients without RA [36]. When treating patients with RA, it is necessary to pay attention not only joint to destruction, but also to osteoporosis. DMB might have many advantages in the treatment of RA because of its ability to inhibit bone erosion and increase BMD.

The results of our sensitivity analysis suggest that the effect of DMB on BMD in patients with RA might be influenced by age. BMD might be increased more in older patients with a longer duration of RA than in middle-aged patients with a shorter duration of RA. Patients with a longer disease duration of RA might be older. It is difficult to exclude the possibility that age and disease duration of RA are confounding factors. The disease activity and inflammatory findings of patients with RA in the selected studies were almost all moderate. Therefore, age and disease duration might not be related to the disease activity or inflammatory condition in this analysis.

Age influenced the variation of BMD in the lumber spine, but not the total hip. It is difficult to explain the reason for this, but we suspect that it might be related to the ratio of trabecular to cortical bone. The percentage of trabecular bone in the lumbar spine and total hip is ∼50% and ∼30%, respectively [34, 37]. DMB is more likely to be effective in trabecular bone. This difference in response to DMB between lumbar spine and total hip might influence the results of sensitivity analysis based on age.

In the present study, we revealed that DMB might decrease JSNS in patients with RA at 12 months after treatment compared with the findings with placebo. This result might suggest that it is possible for DMB to inhibit cartilage destruction. However, the direct effects of DMB on cartilage or chondrocytes have not been determined. In previous studies, metabolic makers of cartilage, such as type II collagen C-terminal telopeptide and cartilage oligomeric matrix protein, decreased after DMB treatment [8, 38]. Additionally, it was indicated that chondrocytes could express RANK, RANKL and osteoprotegerin [39]. In the future, the effect of DMB on cartilage tissue needs to be clarified in order to consider its impact on the pathogenesis of RA.

There were some limitations in this meta-analysis. First, heterogeneity was high in some analyses. One of the reasons was that our study was based on a population with various backgrounds. Some studies lacked some clinical information, such as inflammatory findings, DAS, BMI, percentage of osteoporosis and T-score, at baseline. Although sensitivity analysis and subgroup analysis were performed, it was difficult to unify the patient background completely. Second, the effect of DMB on BMD and the prevention of joint destruction during severe inflammation could not be assessed because the disease activity of RA was moderate in most of the studies selected for this research. Third, it was difficult to rule out the possibility of publication bias. Hence, the results of this study should be interpreted with caution. Fourth, it was difficult to evaluate the background factors of patients with RA affecting the efficacy of DMB in terms of the inhibition of joint destruction by sensitivity analysis owing to the small number of selected studies. It was difficult to evaluate whether the dose of GC and MTX influenced the efficacy of DMB on BMD because there were only a few studies reported the dose of GC and MTX. Finally, DMB did not reduce the fracture risk in patients with RA in our analysis. However, in many studies selected for this analysis, there was no patient with a fracture in groups treated with DMB and treated with placebo or BPs during the observation period. To obtain a more accurate estimate of the effect of DMB on fracture risk in patients with RA, a large cohort study needs to be conducted.

In conclusion, DMB is an interesting drug that inhibits joint destruction and strengthens bones in patients with RA, although it does not improve the disease activity of RA. In the future, a more detailed evaluation is needed to determine whether DMB can suppress joint destruction in patients with persistent high inflammation, who are unable to receive adequate immunosuppressive therapy.

## Supplementary Material

rkab099_Supplementary_Data
