# Supplementary material for: Therapeutic efficacy of denosumab for rheumatoid arthritis: a systematic review and meta-analysis
Source: Rheumatol Adv Pract. 2021 Dec 17;5(3):rkab099. doi: 10.1093/rap/rkab099 (PMC8693364; doi:10.1093/rap/rkab099)
Supplement: rkab099_Supplementary_Data [file rkab099_supplementary_data.docx]

**Supplementary Table S1. Characteristics of included studies in this meta-analysis**

| Study  Author & publish year | Study design | Group | Number | HAQ-DI | DAS28-ESR | CRP  (mg/dl) | Dose of GC  (mg/day) | bDMARDs  (%) | csDMARDs  (%) |
| --- | --- | --- | --- | --- | --- | --- | --- | --- | --- |
| Mochizuki2021 | Cohort | DMB | 112 | NA | NA | NA | NA | 57.1 | NA |
| Mori2021 | Cohort | BPs  DMB | 50  56 | 0.52±0.43  0.64±0.77 | NA  NA | NA  NA | 5.36±1.4  5.4±2.3 | 60  44.6 | NA  NA |
| So2021 | RCT | Placebo  DMB | 55  55 | 0.13[0-0.5]^a^  0.25[0-0.88]^a^ | NA  NA | 0.15[0.07-0.43]^a^  0.17[0.09-0.61]^a^ | NA  NA | NA  NA | NA  NA |
| Tanaka2021 | RCT | Placebo  DMB | 105  191 | NA  NA | NA  NA | 0.4±0.6  0.6±1.3 | NA  NA | NA  NA | NA  NA |
| Tanaka2020 | RCT | Placebo  DMB | 306  302 | NA  NA | NA  NA | 0.47±0.84  0.61±1.16 | NA  NA | NA  NA | NA  NA |
| Hattori2019 | Cohort | DMB | 74 | 0.90±0.84 | NA | 0.78±1.23 | NA | 23 | NA |
| Ishiguro2019 | RCT | Placebo  DMB | 66  70 | NA  NA | NA  NA | NA  NA | NA  NA | NA  NA | NA  NA |
| Kaneko2019 | Cohort | Naïve  Switched | 25  11 | NA  NA | 3.6±0.2  4.2±0.3 | NA  NA | NA  NA | 32  54.5 | 36  27.3 |
| Suzuki2019 | Cohort | DMB  DMB+VD | 31  27 | 0.4±0.1  0.4±0.1 | NA  NA | NA  NA | NA  NA | NA  NA | NA  NA |
| Ebina2018 | Cohort | BPs  DMB | 30  30 | NA  NA | NA  NA | 1.1±0.3  0.6±0.2 | 2.8±0.5  2.8±0.6 | NA  NA | NA  NA |
| Mochizuki2018 | Cohort | DMB | 70 | NA | 2.8±0.9 | 0.26±0.2 | NA | NA | NA |
| Takeuchi2018 | RCT | Placebo  DMB | 218  217 | 0.31±0.39  0.40±0.51 | NA  NA | 0.36±0.57  0.65±1.25 | 3.73±1.89  3.96±2.16 | NA  NA | NA  NA |
| Ebina2017 | Cohort | BPs  DMB | 80  74 | NA  NA | NA  NA | 0.7±0.1  0.6±0.1 | NA  NA | 18.8  29.7 | NA  NA |
| Nakamura2017 | Cohort | DMB  DMB+VD | 22  21 | 0.44±0.2  0.39±0.2 | NA  NA | NA  NA | NA  NA | NA  NA | NA  NA |
| Nakamura2017_2 | Cohort | BPs  DMB | 26  26 | 0.6±0.1  0.7±0.2 | NA  NA | NA  NA | NA  NA | 34.6  30.8 | NA  NA |
| Kinoshita2016 | Cohort | BPs  DMB | 49  49 | NA  NA | NA  NA | NA  NA | NA  NA | NA  NA | NA  NA |
| Takeuchi2016 | RCT | Placebo  DMB | 88  85 | 0.47±0.52  0.39±0.45 | NA  NA | 0.75±1.24  0.52±0.92 | NA  NA | NA  NA | 25  21.2 |
| Dore2009 | Cohort | Placebo_nb  DMB_nb  Placebo_b  DMB_b  Placebo_ng  DMB_ng  Placebo_g  DMB_g | 56  60  22  13  49  45  29  29 | NA  NA  NA  NA  NA  NA  NA  NA | NA  NA  NA  NA  NA  NA  NA  NA | NA  NA  NA  NA  NA  NA  NA  NA | NA  NA  NA  NA  NA  NA  NA  NA | NA  NA  NA  NA  NA  NA  NA  NA | NA  NA  NA  NA  NA  NA  NA  NA |
| Cohen2008 | Cohort | Placebo  DMB | 78  73 | NA  NA | NA  NA | NA  NA | NA  NA | 22  19 | NA  NA |

^a^ 95% CI, DMB: denosumab, BPs: bisphosphonates, VD: Vitamin D, nb: no bisphosphonates, b: bisphosphonatess, ng: no glucocorticoids, g: glucocorticoids, SD: standardized deviation, HAQ-DI: Healthy Assessment Questionnaire-Disability Index, DAS28-ESR: Disease Activity Score 28-Erythrocyte segmentation rate, CRP: C-reactive protein, GC: glucocorticoid, bDMARDs: biologics Disease Modifying Anti-Rheumatic Drugs, csDMARDs: conventional synthetic Disease Modifying Anti-Rheumatic Drugs, NA: not available.

| Author | Criteria  1 | Criteria  2 | Criteria  3 | Criteria  4 | Criteria  5 | Criteria  6 | Criteria  7 | Criteria  8 | Criteria  9 | Criteria  10 | Criteria  11 | Criteria  12 | Criteria  13 | Criteria  14 | Total |
| --- | --- | --- | --- | --- | --- | --- | --- | --- | --- | --- | --- | --- | --- | --- | --- |
| Mochizuki2021 | Yes | Yes | NA | Yes | No | Yes | Yes | No | Yes | Yes | Yes | No | Yes | Yes | 10/14 |
| Mori2021 | Yes | Yes | NA | Yes | No | Yes | Yes | No | Yes | Yes | Yes | No | NA | Yes | 9/14 |
| So2021 | Yes | Yes | Yes | Yes | Yes | Yes | Yes | No | Yes | Yes | Yes | Yes | NA | Yes | 12/14 |
| Tanaka2021 | Yes | Yes | NA | Yes | No | Yes | Yes | No | Yes | Yes | Yes | Yes | NA | Yes | 10/14 |
| Tanaka2020 | Yes | Yes | NA | Yes | No | Yes | Yes | Yes | Yes | Yes | Yes | Yes | NA | Yes | 11/14 |
| Hattori2019 | Yes | Yes | NA | Yes | No | Yes | Yes | Yes | Yes | Yes | Yes | No | NA | Yes | 10/14 |
| Ishiguro2019 | Yes | Yes | NA | Yes | No | Yes | Yes | Yes | Yes | Yes | Yes | Yes | NA | No | 10/14 |
| Kaneko2019 | Yes | Yes | NA | Yes | No | Yes | Yes | No | Yes | Yes | Yes | No | NA | Yes | 9/14 |
| Suzuki2019 | Yes | Yes | NA | Yes | No | Yes | Yes | No | Yes | Yes | Yes | No | NA | No | 8/14 |
| Ebina2018 | Yes | Yes | NA | Yes | No | Yes | Yes | Yes | Yes | Yes | Yes | No | NA | Yes | 10/14 |
| Mochizuki2018 | Yes | Yes | NA | Yes | No | Yes | Yes | No | Yes | Yes | Yes | No | NA | No | 8/14 |
| Takeuchi2018 | Yes | Yes | Yes | Yes | Yes | Yes | Yes | Yes | Yes | Yes | Yes | Yes | NA | Yes | 13/14 |
| Ebina2017 | Yes | Yes | NA | Yes | No | Yes | Yes | Yes | Yes | Yes | Yes | No | Yes | Yes | 11/14 |
| Nakamura2017 | Yes | Yes | NA | Yes | Yes | Yes | Yes | Yes | Yes | Yes | Yes | No | NA | No | 1014 |
| Nakamura2017_2 | Yes | Yes | NA | Yes | Yes | Yes | Yes | No | Yes | Yes | Yes | No | NA | No | 9/14 |
| Kinoshita2016 | Yes | Yes | NA | Yes | No | Yes | Yes | No | Yes | Yes | Yes | No | Yes | No | 9/14 |
| Takeuchi2016 | Yes | Yes | Yes | Yes | Yes | Yes | Yes | Yes | Yes | Yes | Yes | Yes | Yes | Yes | 14/14 |
| Dore2009 | Yes | Yes | NA | Yes | No | Yes | Yes | Yes | Yes | Yes | Yes | No | NA | No | 9/14 |
| Cohen2008 | Yes | Yes | NA | Yes | Yes | Yes | Yes | Yes | Yes | Yes | Yes | Yes | No | No | 11/14 |

**Supplementary Table S2. The Quality Assessment Tool for Observational Cohort and Cross-Sectional Studies from National Heart, Lung, and Blood Institute (NHLBI)**

Criteria 1: Was the research question or objective in this paper clearly stated?

Criteria 2: Was the study population clearly specified and defined?

Criteria 3: Was the participation rate of eligible persons at least 50%?

Criteria 4: Were all the subjects selected or recruited from the same or similar populations (including the same time period)? Were inclusion and exclusion criteria for being in the study prespecified and applied uniformly to all participants?

Criteria 5: Was a sample size justification, power description, or variance and effect estimates provided?

Criteria 6: For the analyses in this paper, were the exposure(s) of interest measured prior to the outcome(s) being measured?

Criteria 7: Was the timeframe sufficient so that one could reasonably expect to see an association between exposure and outcome if it existed?

Criteria 8: For exposures that can vary in amount or level, did the study examine different levels of the exposure as related to the outcome (e.g., categories of exposure, or exposure measured as continuous variable)?

Criteria 9: Were the exposure measures (independent variables) clearly defined, valid, reliable, and implemented consistently across all study participants?

Criteria 10: Was the exposure(s) assessed more than once over time?

Criteria 11: Were the outcome measures (dependent variables) clearly defined, valid, reliable, and implemented consistently across all study participants?

Criteria 12: Were the outcome assessors blinded to the exposure status of participants?

Criteria 13: Was loss to follow-up after baseline 20% or less?

Criteria 14: Were key potential confounding variables measured and adjusted statistically for their impact on the relationship between exposure(s) and outcome(s)?


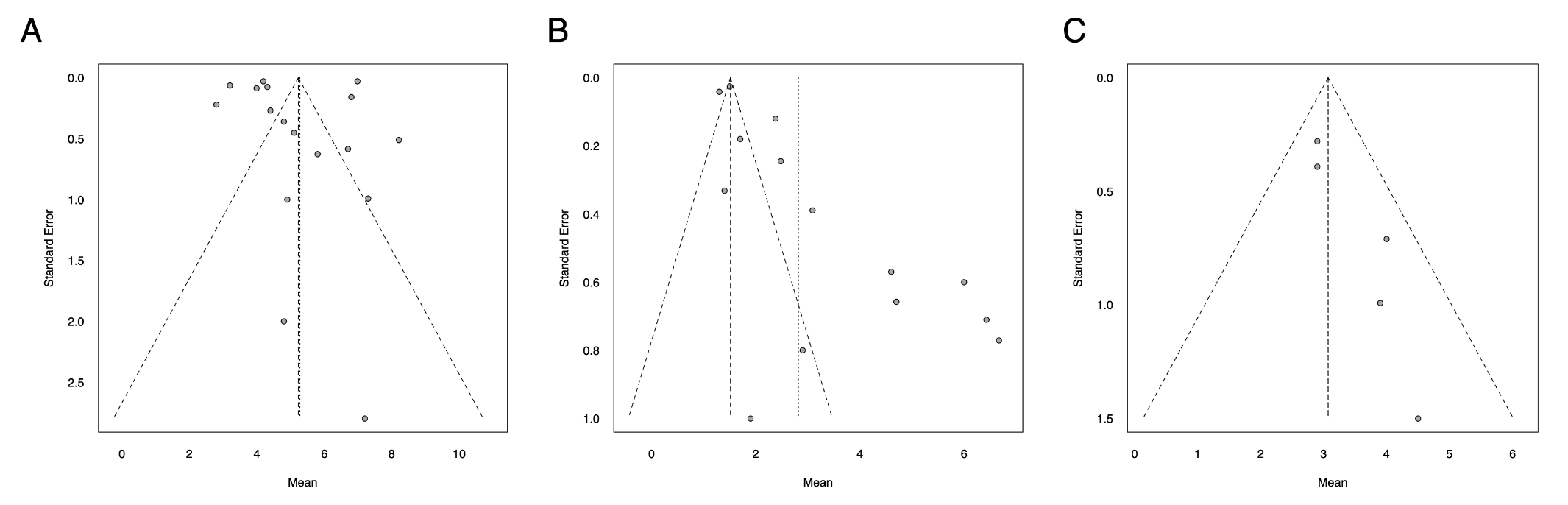


**Supplementary Figure S1. Funnel plot of the effect of denosumab on BMD in patients with RA at 12 months from baseline.** (A) The effect of denosumab on BMD in lumber spine. (B) The effect of denosumab on BMD in total hip. (C) The effect of denosumab on BMD in femoral neck. Each solid circle represents a study. The y-axis represents the standard error that reflects the number of samples, and the x-axis shows the mean rate of change in BMD, which reflects the effect size. Dotted line indicates random effect model estimate and dashed line indicates fixed effect model estimate. The outer dashed lines indicate the triangular region within which 95% of studies are expected to lie in the absence of both biases and heterogeneity. BMD: bone mineral density.


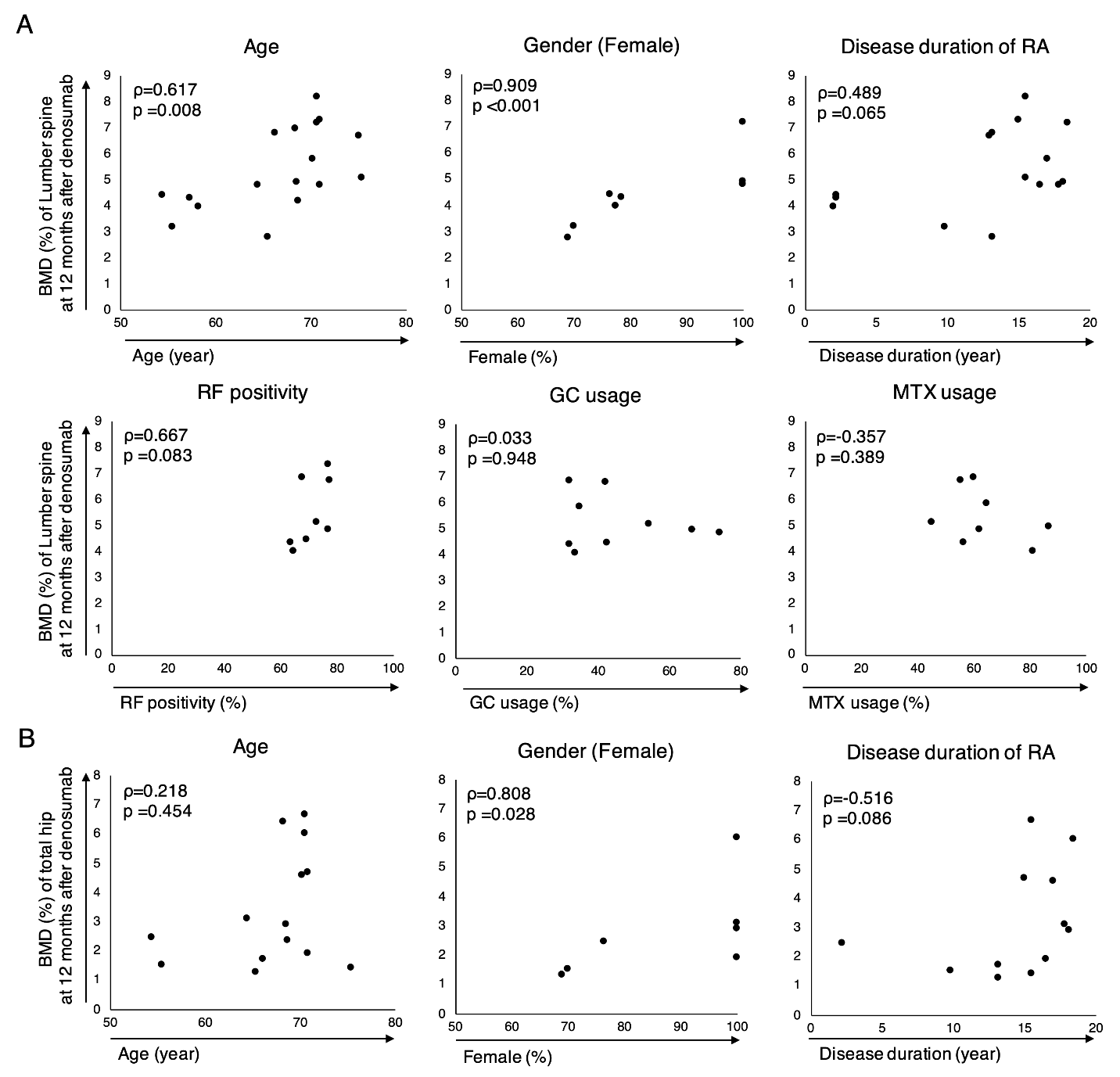


**Supplementary Figure S2. Sensitivity analysis for the effect of denosumab on BMD in lumber spine and total hip at 12 months from baseline.** (A) The correlation between the mean variation rate of BMD in lumber spine at 12 months after denosumab treatment and age, gender, disease duration of RA, the positivity of RF, and the usage of glucocorticoid and methotrexate were evaluated by Spearman’s correlation coefficient. (B) The correlation between the mean variation rate of BMD in total hip at 12 months after denosumab treatment and age, gender, and disease duration of RA were also evaluated. BMD: bone mineral density, RF: rheumatoid factor, RA rheumatoid arthritis.


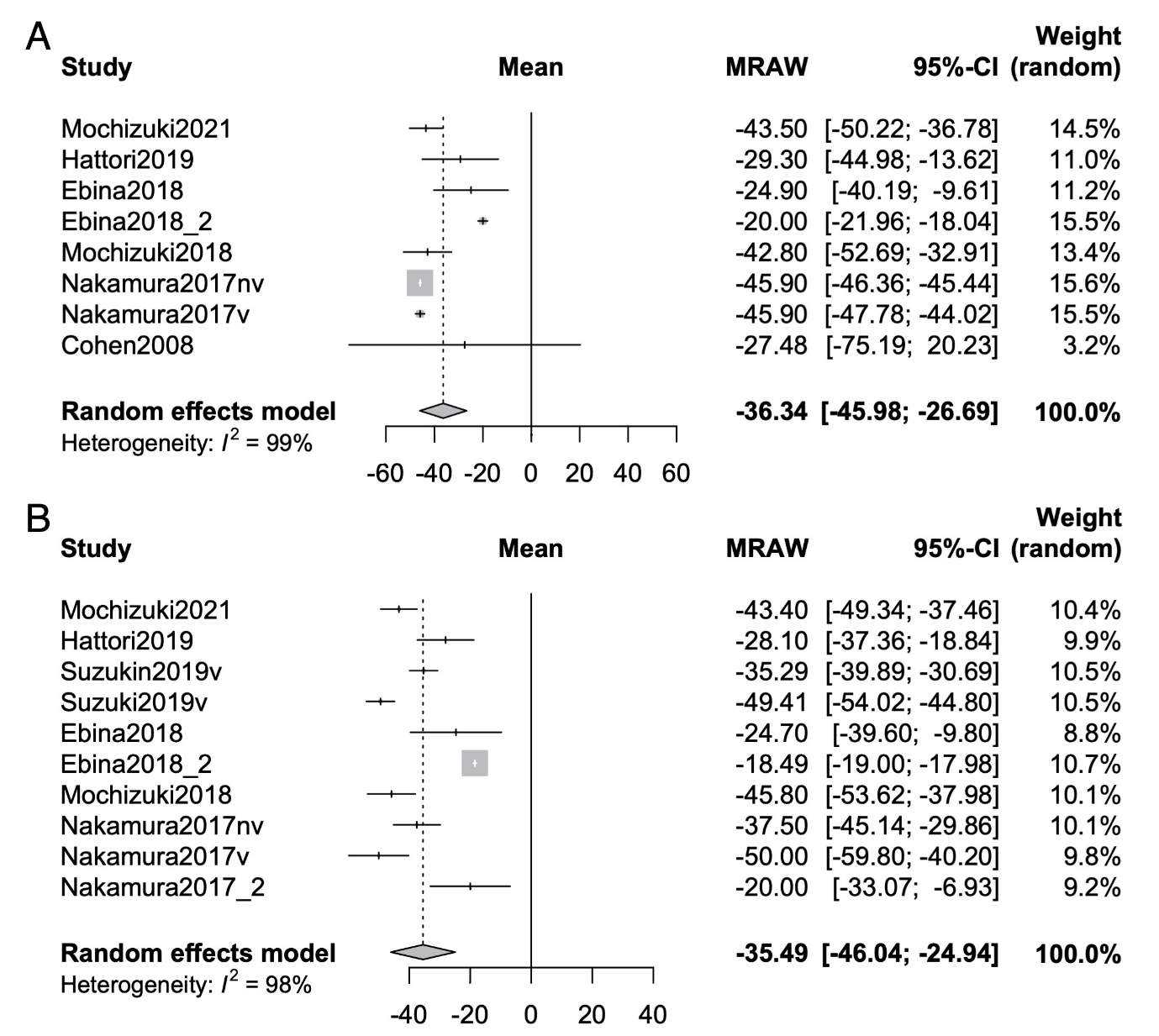


**Supplementary Figure S3. Forrest plot of the variation rate of bone turnover marker in patients with RA at 12 months after denosumab treatment.** (A) The variation rate of P1NP at 12 months from baseline. (B) The variation rate of TRACP-5b at 12 months from baseline. MRAW: mean raw value.


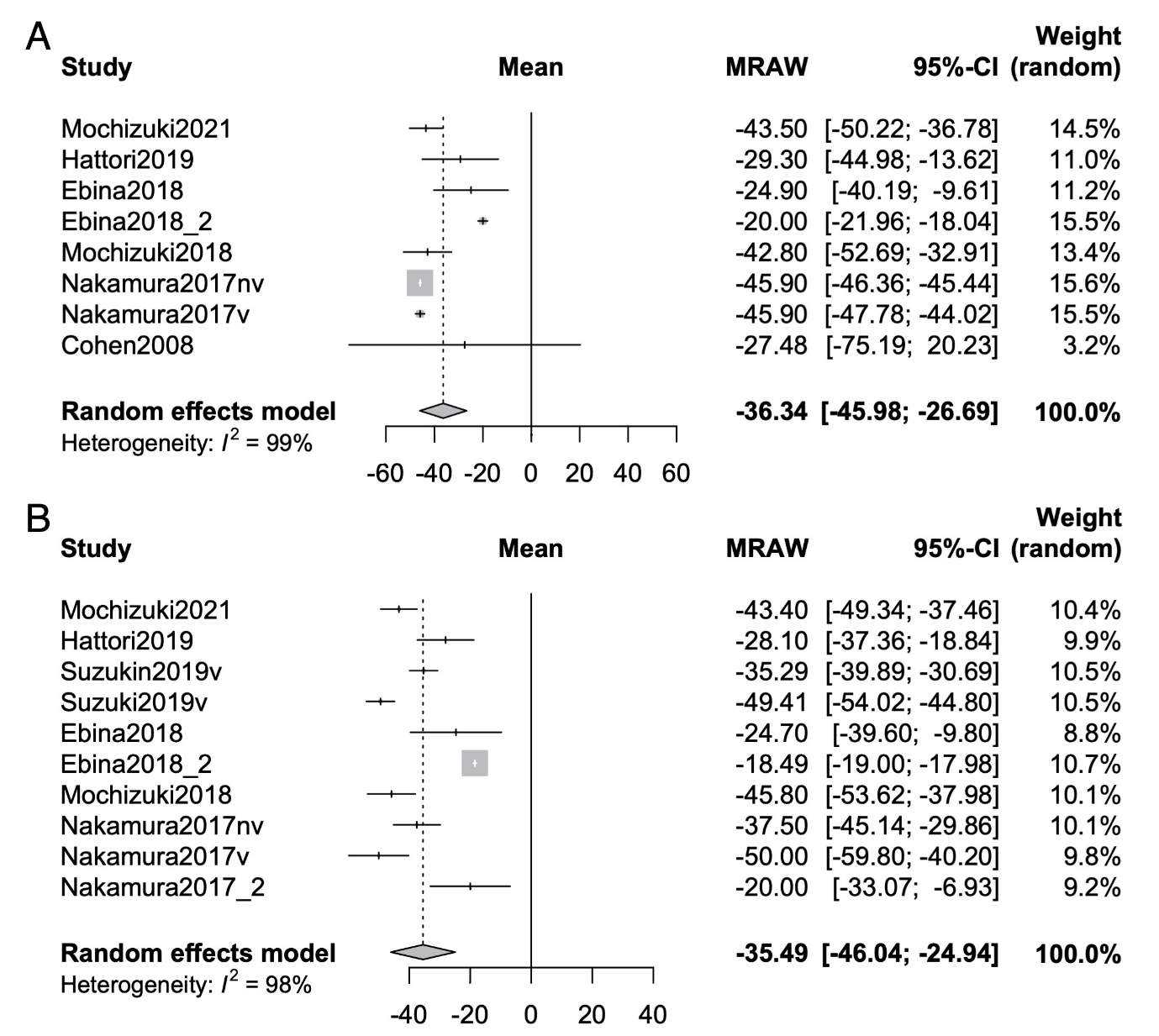


**Supplementary Figure S4. Forrest plot of the variation rate of disease activity score in patients with RA at 12 months after denosumab treatment.** (A) The variation rate of DAS28-ESR at 12 months from baseline. (B) The variation rate of SDAI at 12 months from baseline. SD: standardized deviation.


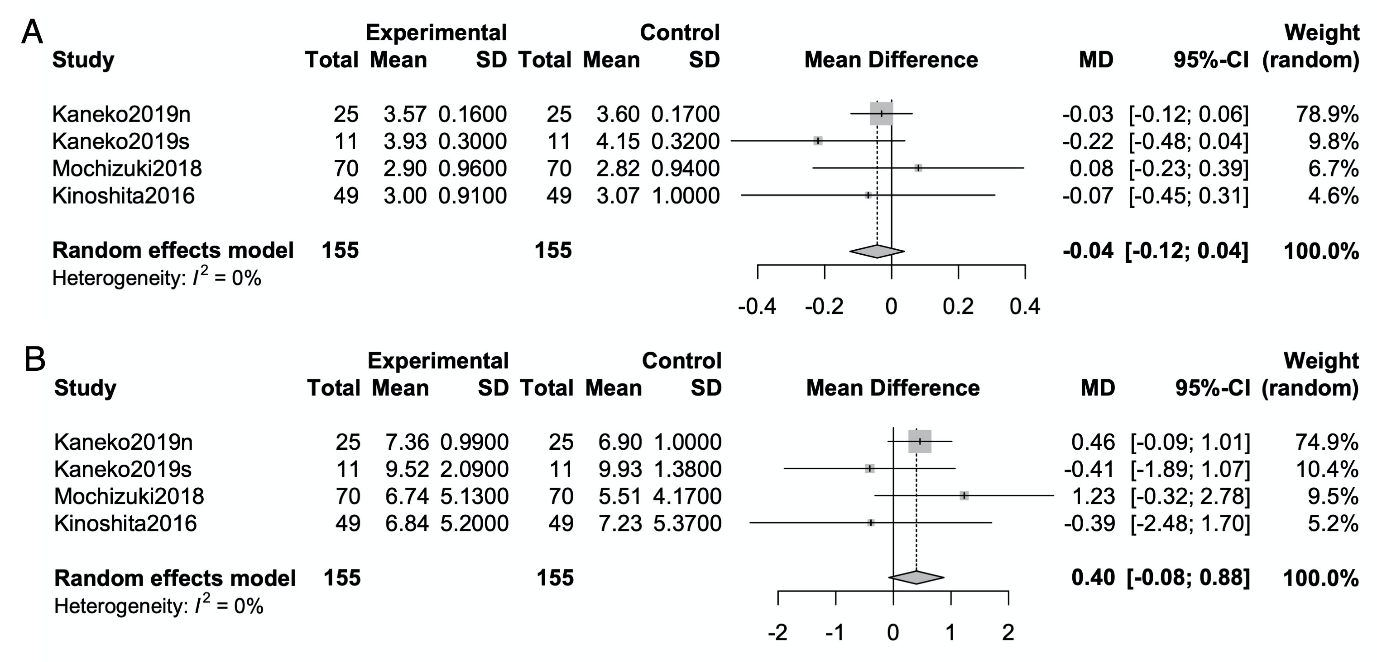


**Supplementary Figure S5. Forrest plot of the risk of fracture at 12 months from baseline between patients with RA treated with denosumab and those treated with bisphosphonates.** RR: relative risk.


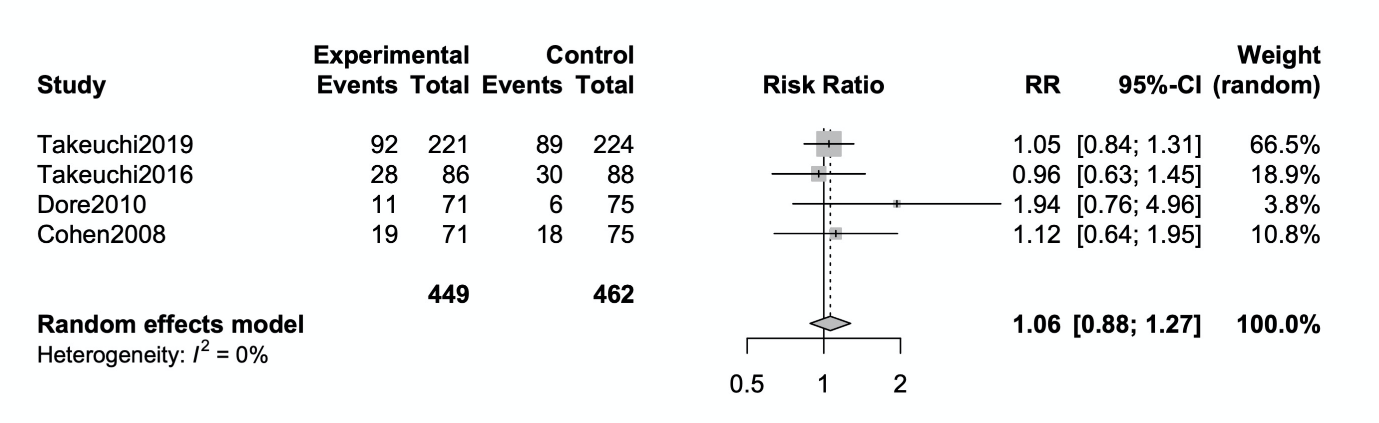


**Supplementary Figure S6. Forrest plot of the risk of upper respiratory infection between patients with RA treated with denosumab and those with placebo.**
